# Changing perspectives: The development of preservice teachers' field‐specific ability beliefs across academic disciplines

**DOI:** 10.1111/bjep.70019

**Published:** 2025-08-13

**Authors:** Katharina Asbury, Bastian Carstensen, Uta Klusmann

**Affiliations:** ^1^ Department of Educational Research and Educational Psychology IPN—Leibniz Institute for Science and Mathematics Education Kiel Germany; ^2^ Humboldt University Berlin Germany

**Keywords:** field‐specific ability beliefs, growth mindset, longitudinal analysis, preservice teachers, STEM, teacher education

## Abstract

**Background:**

Field‐specific ability beliefs (FABs) reflect the perception that success in academic fields depends on innate, unteachable abilities. These beliefs affect teaching practices and student motivation. However, little is known about their longitudinal development during teacher education.

**Aims:**

This study examines how FABs evolve during university teacher education and whether longitudinal trajectories differ across academic disciplines, particularly between STEM and non‐STEM subjects.

**Sample:**

The sample included 1734 preservice teachers (22.11 years old on average in the first study year, 69.8% female) from a German university, studying across 21 subjects, categorized into six groups: languages; arts and philosophy; social sciences; physical education; biology, chemistry and computer science and mathematics‐intensive subjects.

**Methods:**

FABs were measured annually over four years using a validated scale. Applying latent growth modelling, we examined overall changes and differences in trajectories across subject domains, controlling for gender and prior academic achievement.

**Results:**

FABs changed over time, with five of the six subject groups showing a decline. Mathematics‐intensive subjects and physical education exhibited the highest initial FAB levels. While mathematics‐intensive subjects showed the steepest decline, FABs in physical education increased. FABs in social sciences and languages remained relatively stable. Gender predicted initial FAB levels but was unrelated to changes over time. Prior achievement did not predict either initial levels or longitudinal changes in FABs.

**Conclusions:**

Teacher education appears to reduce FABs, particularly in STEM fields, yet persistent and increasing FABs in certain disciplines highlight the need for targeted interventions to foster growth‐oriented beliefs in preservice teachers.

## INTRODUCTION

Teachers have a substantial effect on student learning and motivation (e.g., Hattie, 2023; Jansen et al., 2022). This influence derives from their professional knowledge, motivation and beliefs, which all shape their instructional behaviour (Kunter et al., 2013). Among these beliefs, field‐specific ability beliefs (FABs) have gained attention as they are associated with student motivation and achievement (Heyder et al., 2019). FABs refer to the belief that success in a particular academic field requires an inherent ability that cannot be taught (Leslie et al., 2015). This concept is related to Dweck's (1999, 2006) mindset theory, which differentiates between growth (abilities are malleable) and fixed (abilities are stable) mindsets. Despite their conceptual overlap, empirical studies have confirmed that FABs and fixed mindsets are distinct constructs (Asbury et al., 2023; Limeri et al., 2023; Porter & Cimpian, 2023).

Prior research has demonstrated that teachers' FABs influence students' motivation and learning outcomes, particularly in STEM subjects (Heyder et al., 2019). In STEM fields, talent is valued more highly than in non‐STEM fields (Leslie et al., 2015; Meyer et al., 2015), which, according to initial findings, may translate into teachers holding stronger FABs in these domains (Asbury et al., 2023; Heyder et al., 2019). Despite growing evidence on the relevance of FABs (Heyder et al., 2019; Meyer et al., 2015), research on their longitudinal development—especially during teacher university training, a formative stage for teachers' beliefs and competencies—is still scarce (Rissanen et al., 2019). Moreover, systematic investigations of FABs in non‐STEM subjects remain rare (Bian et al., 2018). By including a broad range of academic disciplines, our study not only addresses this gap but also explores whether FABs show distinct developmental patterns across STEM and non‐STEM domains.

We aim to address key gaps in current research on FABs, particularly concerning their development during teacher education. Specifically, we investigate whether FABs change over the course of university teacher education after entering university and how their development varies depending on the subject studied. To gain insights into these processes, we analysed a longitudinal dataset of 1734 preservice teachers across 21 subjects over four years of teacher university education.

### Teacher beliefs as part of professional competence

Teachers' beliefs are a central component of their professional competence and play a crucial role in shaping instructional practices and student outcomes (Fives & Buehl, 2012; Kunter et al., 2013). According to the COACTIV model (Baumert et al., 2013), teacher competence consists of professional knowledge, motivational orientations, self‐regulation and beliefs. Among these components, teacher beliefs influence expectations about student learning, approaches to instruction and classroom interactions (Rubie‐Davies, 2008). Research has demonstrated that teachers' beliefs about intelligence and learning impact their classroom practices, which in turn affect student motivation and achievement (Canning et al., 2019; Eccles & Wigfield, 2020). Specifically, beliefs about the nature of academic ability—whether it is fixed or malleable—can shape how teachers assess students' potential and respond to challenges in the classroom (e.g., Rattan et al., 2012).

Within this broader framework of teacher beliefs, field‐specific ability beliefs (FABs) have emerged as a particularly relevant construct, as they reflect perceptions of the role of inherent ability (“brilliance”) in determining success in different academic fields (Leslie et al., 2015). These beliefs are not only theoretically linked to mindset theory (Dweck, 2006) but also have significant practical implications: teachers who hold strong FABs may reinforce domain‐specific stereotypes about student ability, which can influence students' motivation, self‐concept and achievement (Heyder et al., 2019). While FABs and mindsets both relate to beliefs about the nature of ability, they differ in important ways. Mindset theory (Dweck, 1999) describes individuals' beliefs about the malleability of their own abilities, typically measured across broad domains such as intelligence. In contrast, FABs reflect socially shared perceptions about what it takes to succeed in specific academic fields, namely, whether achievement in a given field requires innate brilliance. Thus, while both constructs address implicit theories of ability, mindsets are domain‐general and self‐focused, whereas FABs are field‐specific and socially embedded. Prior research demonstrates a clear empirical distinction between the two constructs (Asbury et al., 2023; Limeri et al., 2023; Porter & Cimpian, 2023), suggesting they capture distinct psychological phenomena.

In the present investigation, we focus on FABs because our research question centres on how preservice teachers perceive discipline‐specific success criteria, a core component of FABs but not of mindset theory. Given the potential consequences of teachers' beliefs for classroom environments, it is essential to understand how FABs develop during teacher education and to what extent they change over time.

### Field‐specific ability beliefs: Theoretical and empirical underpinnings

FABs represent a specific dimension of teachers' professional beliefs, reflecting the extent to which success in an academic domain is perceived to depend on innate ability rather than effort (Leslie et al., 2015). High FABs in teachers have been shown to negatively affect instructional practices and student outcomes (Heyder et al., 2019). For instance, they may contribute to self‐fulfilling prophecies, lower expectations and reduced motivation among students perceived as less “naturally gifted” (Bian et al., 2018; Canning et al., 2019). FABs are particularly prevalent in STEM fields, where cultural narratives often emphasize brilliance as a prerequisite for success (Leslie et al., 2015; Meyer et al., 2015). These beliefs can reinforce participation gaps and influence how teachers assess student potential. While FABs conceptually overlap with intelligence mindsets (Dweck, 2006), research suggests they represent related but empirically distinct constructs (Asbury et al., 2023; Limeri et al., 2023; Porter & Cimpian, 2023). Despite their importance for career prospects (Cachaper et al., 2008; Van Aalderen‐Smeets et al., 2019), many students, particularly girls and women, evaluate STEM careers as unappealing (Blotnicky et al., 2018; Wang & Degol, 2017). Globally, STEM workforce shortages persist, with 40% of EU employers struggling to fill related roles (CEDEFOP, 2020). If teachers believe that success in STEM subjects is largely based on talent, this could further discourage students already struggling with these subjects. Understanding teachers' FABs, their origins, subject‐specific differences and developmental trajectories is crucial to addressing this issue.

While most research on FABs focuses on STEM fields, emerging work suggests that FABs also shape perceptions in non‐STEM domains, albeit with different emphases. In physical education, for instance, success is often linked to physical aptitude rather than cognitive brilliance. Discourses around natural athleticism and talent identification are central to sports science and PE pedagogy (Croston, 2012; Williams & Reilly, 2000), fostering beliefs that certain students are “born athletes”. These reflect fixed‐ability beliefs similar to those found in STEM, but centred on physical rather than intellectual traits. Likewise, in the arts, talent is frequently framed as an innate gift—something one either possesses or does not. Research in arts education shows that such views can affect student motivation and teacher expectations (Oreck, 2004; Winner & Martino, 2000), with teachers often seeing artistic ability as “untrainable” and offering less support to students lacking early signs of talent. These cultural narratives surrounding physical and creative giftedness may help explain why FABs in fields like PE and the arts follow different developmental patterns than in other disciplines. However, systematic research on FABs in these areas remains limited, pointing to a need for further study.

### Gender differences in FABs

Prior research suggests gender differences in FABs, particularly in STEM domains. For example, studies have shown that male teachers are more likely to endorse beliefs linking success in mathematics or science to innate talent (Heyder et al., 2019), while female teachers often emphasize the role of effort and learning strategies (Leslie et al., 2015). These patterns align with broader gender stereotypes about intellectual ability—such as the association of “brilliance” with males—which can shape both self‐perceptions and beliefs about others (Bian et al., 2018; Leslie et al., 2015; Meyer et al., 2015; Storage et al., 2016). For instance, Leslie et al. (2015) found that academic fields emphasizing innate talent over effort tend to have lower female representation, a pattern linked to the stereotype that brilliance is more characteristic of men than women. Similarly, Bian et al. (2018) showed that as early as age six, girls are less likely than boys to associate high‐level intellectual ability with their own gender. Such findings suggest that gendered beliefs about ability are both pervasive and internalized, potentially shaping individuals' FABs. These documented patterns provide theoretical support for including gender as a covariate in our analysis.

### The development of FABs in university students: Insights from mindset research

Substantial research has explored the development of intelligence mindsets (Dweck, 2006). Longitudinal studies suggest that beliefs about ability are not static and may change throughout university education. However, findings are mixed and appear to depend on both context and the specificity of the measures used. Some studies assessing general intelligence mindsets suggest stability over time. Robins and Pals (2002) found no significant change in fixed mindsets over two years among college students; individual differences remained stable. In contrast, Stephens et al. (2021) observed a shift towards a growth mindset among preservice teachers over a similar period, potentially due to exposure to pedagogical training that emphasizes learning as a developmental process.

Beyond general mindsets, several studies have examined beliefs about ability in specific academic domains, closely aligned with FABs. Findings suggest that while general intelligence mindsets may remain stable or shift towards growth orientations, domain‐specific beliefs, especially in STEM fields, tend to become more fixed over time. For instance, studies in mathematics, biology, chemistry and computer science consistently show that students develop more fixed beliefs about domain‐specific abilities as they progress through university education (e.g., Dai & Cromley, 2014; Flanigan et al., 2017; Limeri et al., 2020; Scott & Ghinea, 2014; Shively & Ryan, 2013). This trend is particularly pronounced among students who struggle academically (Limeri et al., 2020). The structure of STEM education—emphasizing competitive grading, high‐stakes assessments and talent‐oriented narratives—may reinforce the perception that success depends on innate aptitude rather than effort (Limeri et al., 2020; Shively & Ryan, 2013). These patterns underscore the need to study FAB development in teacher education, where beliefs about ability may be reshaped by pedagogical training or practical classroom experiences.

### The German teacher education system

Germany's teacher education is highly standardized and consists of three phases: (1) a three‐year Bachelor of Education programme, (2) a two‐year Master of Education programme and (3) an in‐service training phase (Cortina & Thames, 2013). The bachelor's programme combines subject‐specific coursework with educational sciences and practical teaching internships, providing preservice teachers with both theoretical knowledge and practical experience. These internships expose preservice teachers to classroom experiences where they observe students' learning processes and may adjust their beliefs about the nature of academic ability.

Given these structured learning opportunities, FABs may decrease over time as preservice teachers become more aware of how instructional factors, such as quality of teaching and student motivation, contribute to learning success. This aligns with previous research on the malleability of teacher mindsets and beliefs during early‐career training (Jonsson & Beach, 2012; Stephens et al., 2021).

## RESEARCH QUESTIONS AND HYPOTHESES

Since teacher education is supposed to enhance teachers' competence, this is a sensitive stage in a teachers' career. In light of previous findings, it is a very suitable time to examine preservice teachers' attitudes and beliefs as they grow into teachers. Hence, this study examines the longitudinal development of preservice teachers' FABs over four years in university teacher education and explores discipline‐specific differences.

First, we investigated whether FABs generally change over the course of university teacher education. Given that teacher education might foster more growth‐oriented perspectives (see Stephens et al., 2021), we hypothesize that FABs will diminish over time (Hypothesis 1). As preservice teachers observe their own learning progress and gain theoretical and practical knowledge, they may come to recognize the role of effort and instructional quality in student success.

Second, we examined potential differences across academic disciplines at the beginning of university teacher education as well as in longitudinal FAB trajectories. Previous research has shown that FABs tend to be higher in STEM subjects compared to non‐STEM fields at both the university and school levels (Heyder et al., 2019; Leslie et al., 2015). Accordingly, we hypothesize that FABs will be strongest among preservice teachers in STEM subjects at the beginning of their training (Hypothesis 2a). However, given the anticipated overall decline in FABs, we further expect the extent and pace of change to vary across subject groups (Hypothesis 2b).

To explore discipline‐specific patterns, we categorized preservice teachers into six subject groups (Destatis, 2024): (1) languages, (2) arts and philosophy, (3) social sciences, (4) physical education, (5) biology, chemistry and computer science and (6) mathematics‐intensive subjects (i.e., mathematics and physics). We controlled for *gender* and *prior academic achievement to ensure robust conclusions*, as these factors may influence FAB levels. Gender is a well‐established variable affecting beliefs about ability, particularly in STEM contexts (Wang & Degol, 2017); prior achievement may shape preservice teachers' beliefs about the role innate abilities play for learning and success in their subjects.

## METHOD

### Participants and procedure

We used data from four waves (2020–2023) of the Student Teacher Professional Development Study (StePs study; Carstensen et al., 2023; see also Carstensen & Klusmann, 2020). StePs is a longitudinal panel of preservice teachers at a large German University, tracking their professional development during university teacher training by assessing individual characteristics as well as contextual factors. For each wave, data collection took place during the winter term, and the multi‐cohort sequential study design allowed new participants (e.g., first‐year students) to enter the panel in each survey wave. StePs was part of the “quality initiative for teacher training” program, which was established in 2015 and was funded by the German Federal Ministry of Education and Research (BMBF); Grant 01JA1923. The study was conducted in accordance with the Declaration of Helsinki and the ethical guidelines of the German Psychological Society (DGPs) and the American Psychological Association (APA). Informed consent was obtained from all participants. Participants were approached during their university courses or contacted via email. Participation was entirely voluntary, and individuals could opt to discontinue their involvement at any point. Furthermore, participants had the option to withdraw their consent by providing their individual pseudonymized ID.

The sample included *N* = 1734 preservice teachers (69.8% female) who participated in at least one measurement occasion. Their average age at their first participation was 22.11 years (*SD* = 2.94). To prepare the data for analysis, we aligned it with the participants' study progression. Specifically, the data were structured so that the first measurement point corresponds to the first year of study, the second to the second year, and so on. This allowed us to track students' FABs over a period of up to four years in university teacher education.

### Measures

#### Field‐specific ability beliefs

We assessed FABs using three items (Leslie et al., 2015; German adaptation by Heyder et al., 2020). Participants rated their agreement with statements about what is required for students to succeed in their subject (e.g., “If students in school want to succeed in [subject], hard work alone won't cut it; they need to have an innate gift or talent”). Responses were given on a seven‐point scale (1 = *strongly disagree* to 7 = *strongly agree*) for both of their subjects. Higher FAB scores indicate a stronger belief in the necessity of innate ability for success. The scale demonstrated good internal consistency across study years (.80 < *α* < .85).

To ensure comparability of FAB scores over time, we tested measurement invariance using confirmatory factor analysis. The results supported configural and metric invariance across all four study years and provided strong overall evidence for scalar invariance, justifying the comparison of latent means over time. When adding gender as a grouping factor, model fit significantly decreased between the metric and scalar invariance models; however, overall fit remained acceptable to good. Additional analyses across academic domains showed that metric and scalar invariance were largely supported at Years 2 and 3, while some deviations emerged at the beginning and end of the study (Years 1 and 4), suggesting minor variation in item functioning across disciplines at these time points. A detailed description of the measurement invariance procedures and results is provided in the Supporting Information.

#### Dummy variables for the subject groups

To examine discipline‐specific patterns in FABs, we categorized the 21 subjects offered in the university teacher education program into six subject groups (see Table 1). This categorization was based on the International Standard Classification of Education (ISCED; UNESCO, 2006) and the subject classification scheme of the German Federal Statistical Office (Destatis, 2024), both of which offer structured frameworks for organizing academic disciplines. The ISCED, developed by UNESCO, provides an internationally comparable system for classifying educational programs and fields of study, facilitating cross‐national educational research and policy analysis. Similarly, the Destatis classification reflects the structure of the German education system and is grounded in both curricular logic and empirical analyses of subject clusters, considering shared content domains, pedagogical approaches and institutional arrangements. Drawing on these classifications allows for a theoretically sound and internationally recognizable grouping of subjects. Given our focus on STEM‐related differences, we treated mathematics‐intensive subjects (mathematics and physics) as the reference category. To account for subject‐specific trajectories, we created five dummy variables, each representing one of the remaining subject groups.

**TABLE 1:** Descriptive statistics of field‐specific ability beliefs (FABs) by subject groups in the first study year.

Label subject group	*N*	Subjects included	FABs	
			*M*	*SD*
Languages	1280	German, English, French, Spanish, Greek, Latin, Russian, Danish, Italian	2.66	1.06
Art and philosophy	273	Art, philosophy	2.92	1.26
Social sciences	942	History, economics, religious education, geography	2.01	.95
Physical education	153	Physical education	3.27	1.30
Biology, chemistry, computer science	492	Biology, chemistry, computer science	2.28	1.04
Mathematics‐intensive subjects	323	Mathematics, physics	3.31	1.42
–	3463	All 21 subjects	2.55	1.19

*Note*: *N* = 3463 subjects reported by the 1734 preservice teachers (in five cases no information on the subjects was available). Descriptive statistics refer to the measurements in the first year of university studies.

#### Covariates

##### Gender

Gender was coded based on the information provided by preservice teachers during their initial participation in the panel (0 = *female*, 1 = *male*, 2 = *nonbinary*). Four participants (.2%) chose not to disclose their gender, and six (.3%) identified as nonbinary. Due to the small sample size of the latter group, these cases were treated as missing values in the analyses. Gender was subsequently included as a dummy variable in all relevant statistical models.

##### Prior achievement

We included participants' upper secondary school leaving certificate grade as an indicator of prior achievement. Grades ranged between 1 (*highest*) and 4 (*lowest*, passing grade). Although not domain‐specific, these grades represent a standardized and psychometrically validated measure of school performance in Germany. In the context of FABs, prior academic achievement may shape individuals' beliefs about the role of talent versus effort. For instance, students who have experienced success with relatively little effort may be more likely to attribute achievement to innate ability, whereas those who link success to persistence and hard work may develop more malleable ability beliefs. Supporting this idea, there is prior evidence that lower‐achieving university students are more likely to hold less adaptive, fixed ability beliefs (e.g., Limeri et al., 2020). We therefore included upper secondary school grades as a covariate to account for participants' academic background at the outset of teacher education. University GPA was available only for a small and selective subgroup of participants and was not included to avoid substantial missingness and selection bias.

#### Analytic strategy

All analyses were conducted with R, Version 4.1.2 (R Core Team, 2022). As a first step, we tested the measurement invariance of the FAB reports to ensure that scores could be meaningfully compared over time. Next, we estimated latent growth models (LGMs), incorporating gender and prior achievement as covariates. Specifically, we tested (a) an intercept‐only model, (b) a model including intercepts and a linear slope and (c) a model that additionally included subject groups (dummy coded) to examine whether these were associated with differential development over time.

#### Latent growth models

To examine the development of FABs over time, we employed LGMs, incorporating gender and prior achievement as covariates (see Figure 2). LGMs decompose variance in repeated measurements into latent intercept and slope factors, which capture both initial levels and systematic changes over time (Burant, 2016).

We first specified an intercept‐only model, also referred to as the stability model (Preacher et al., 2008), which assumed that FABs remained stable over time, meaning no systematic change occurred. This model served as a baseline for evaluating whether a growth trajectory existed. Next, we tested a slope model, which introduced a linear slope to assess whether FABs exhibited systematic increases or decreases across the four study years. This model allowed us to determine whether preservice teachers' FABs changed over time rather than remaining constant. Finally, we specified a conditional growth model, which incorporated subject groups (dummy‐coded) to examine whether subjects were associated with differences in FAB trajectories.

Since preservice teachers in Germany are required to study at least two subjects and students reported their FABs for both, our data had a multilevel structure (subjects nested within individuals). Before analysis, we transformed the dataset into a long format, creating two rows per participant and using the individual student ID as a clustering variable. This adjustment accounted for the dependency of observations and corrected for potential sampling error.

### Missing data

Longitudinal studies frequently encounter challenges related to panel attrition, where participants temporarily or permanently drop out of the study (Wooden, 2001). In addition to this common issue, the multi‐cohort sequential design of the present study introduced additional missing data. Because all currently enrolled preservice teachers were invited to participate at each measurement occasion, some participants had missing values for certain waves due to their later entry into the study. As a result, a relatively high proportion of participants provided data at only a single measurement occasion (*n*‐ = 1273, 73.4%). Nevertheless, sufficient data were available for each study year, with 788 responses on FABs of preservice teachers in the first study year, 464 in the second, 621 in the third and 520 in the fourth.

To examine potential selection effects, we analysed whether participation patterns were systematically related to demographic variables and FABs. Female preservice teachers participated in more measurement occasions than their male counterparts; *χ* ^2^(3) = 56.53, *p* < .001, Cramer's *V* = .13, though this effect was small. Additionally, there was a small but statistically significant effect of prior achievement on participation rates (*p* < .001, Cohen's *f* = .12). Importantly, the number of measurement occasions attended was not statistically significantly associated with FABs (*p* = .190, Cohen's *f* = .03), suggesting that missing data were unlikely to bias the main analyses.

To handle missing data, we applied full information maximum likelihood (FIML) estimation, which produces less biased and more efficient estimates than traditional approaches such as listwise deletion (Collins et al., 2001; Enders & Bandalos, 2001; Little & Rubin, 2019).

## RESULTS

**FIGURE 1:**
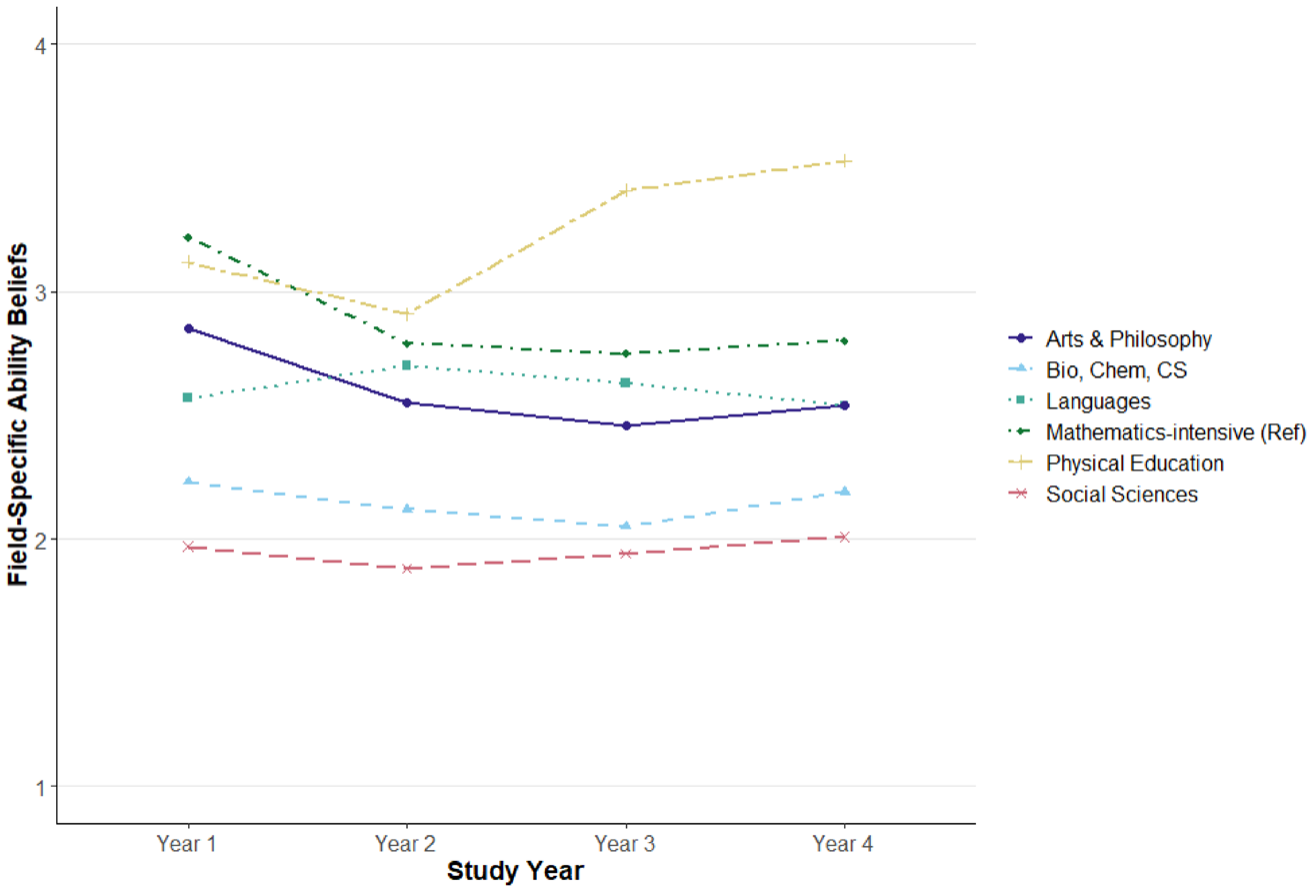
FABs in the six subject groups over the course of university studies. The scale ranges from 1 (= *strongly disagree*) to 7 (= *strongly agree*); for readability, only the range from 1 to 4 is displayed.

Figure 1 provides an overview of preservice teachers' average FABs per subject group over the four study years. A detailed overview of FABs for each of the 21 subjects is presented in Figure S1. In the first study year, FABs across all subjects were relatively low, falling below the theoretical midpoint of the 1 to 7 scale (*M* = 2.55, *SD* = 1.19). However, substantial differences emerged between subject groups (see Table 1). Table 2 summarizes the fit statistics for the different LGMs. While all models demonstrated sufficient fit, the results of the *χ* ^2^‐difference test (*χ* ^2^(5) = 9.59, *p* = .088) indicated a slightly worse fit of the intercept‐only model when compared to the slope model. This indicates substantial changes in FABs over time. In the following, we will report the results that apply to the conditional growth model, including covariates as well as subject groups as predictors of intercept and slope (for an overview, see Table 3).

**TABLE 2:** Model fit statistics of the latent growth models.

Model	*χ* ^2^	*df*	Fit indices			
			CFI	TLI	RMSEA	SRMR
Intercept	155.79	67	.99	.98	.02	.06
Slope	144.77	62	.99	.98	.02	.05
Full	291.16	112	.97	.96	.02	.04

*Note*: The intercept‐only model assumed no change in FABs. The slope model assumed a linear change in FABs. The full model included the subject dummies as predictors for intercept and slope. All models included the covariates gender and prior achievement.

Abbreviations: CFI, comparative fit index; RMSEA, root mean square error of approximation; SRMR, standardized root mean square residual; TLI, Tucker–Lewis index.

**TABLE 3:** Parameter estimates of the slope and conditional growth models.

Predictors	Slope model		Conditional growth model	
	Intercept	Slope	Intercept	Slope
	*B* (SE)	*B* (SE)	*B* (SE)	*B* (SE)
Covariates				
Gender	.**14** (.07)	−.04 (.04)	.**16** (.07)	−.03 (.04)
Prior achievement	−.05 (.06)	.03 (.04)	−.01 (.06)	.02 (.04)
Subject groups				
Languages			**−.56** (.13)	.**14** (.06)
Art & philosophy			**−.43** (.16)	.02 (.09)
Social sciences			**−1.44** (.13)	.**19** (.06)
Physical education			−.01 (.20)	.**33** (.11)
Biology, chemistry, computer science			**−1.11** (.14)	.**16** (.07)

*Note*: *B*‐values represent unstandardized estimates on the standardized intercept and slope factors. Subject group coefficients indicate differences in FAB intercept and slope relative to the reference group (mathematics‐intensive subjects). Statistically significant coefficients (*p* < .05) appear in bold.

**FIGURE 2:**
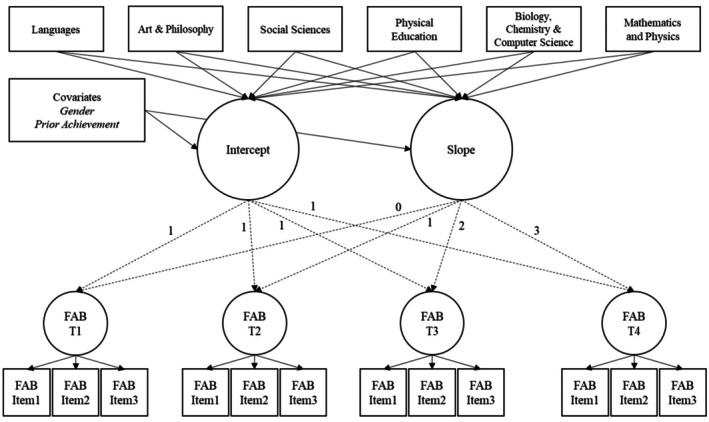
Latent growth model for FABs. Subjects were dummy‐coded with mathematics and physics as the reference category. To maintain clarity and overview, detailed specifications of the scalar measurement invariance (e.g., correlated residuals) are not displayed.

### Initial FABs and trajectories over the course of university teacher education

The latent intercept, representing preservice teachers' FAB scores in the first study year, showed statistically significant variation. This variation was partially explained by gender, with male preservice teachers reporting statistically significant stronger FABs (*B* = .16, *p* = .033). In contrast, prior achievement was not associated with the intercept (*B* = −.01, *p* = .926). Variance in the slope factor was not explained by either gender (*B* = −.03, *p* = .479) or prior achievement (*B* = .02, *p* = .517), suggesting that changes in FABs over time were independent of these individual characteristics. Notably, the remaining variance in the slope factor was not statistically significant, indicating that FAB trajectories over time did not differ substantially across individuals once subject group and covariates were accounted for. This suggests a relatively uniform developmental pattern within groups, with little evidence for individual deviations from the group‐level trends.

### Differential changes in FABs between subject groups

The latent intercept varied significantly across subject groups, with preservice teachers in mathematics‐intensive subjects reporting high initial FABs (*M* = 3.22, *SD* = 1.34), which were comparable to those in physical education (*M* = 3.12, *SD* = 1.23; *B* = −.01, *p* = .980). No statistically significant difference was observed between these two groups, indicating similarly strong fixed‐ability beliefs in both domains. Compared to this group, all other subject groups exhibited significantly lower FABs in the first study year. The strongest differences were observed for social sciences (*M* = 1.97, *SD* = .87; *B* = −1.44, *p* < .001) and biology, chemistry and computer science (*M* = 2.23, *SD* = .97; *B* = −1.11, *p* < .001), followed by languages (*M* = 2.57, *SD* = .99; *B* = −.56, *p* < .001) and art/philosophy (*M* = 2.85, *SD* = 1.18; *B* = −.43, *p* = .007). In contrast, initial FABs in physical education (*M* = 3.12, *SD* = 1.23) did not differ significantly from those in mathematics‐intensive subjects (*B* = −.01, *p* = .980).

The slope factor revealed distinct developmental patterns across subject groups. Mathematics‐intensive subjects, which served as the reference group in the model, exhibited a notable decrease from the first study year (*M* = 3.22, *SD* = 1.34) to the fourth (*M* = 2.80, *SD* = 1.05), corresponding to a raw decline of .42 points on the 7‐point scale. This change reflects an approximate effect size of *d* = −.35, indicating a small‐to‐moderate downward trend. In comparison, FABs in social sciences remained relatively stable over time (first year: *M* = 1.97, *SD* = .87; fourth year: *M* = 2.01, *SD =* .93), with a small positive standardized slope (*B* = .19, *p* = .003). FABs in physical education increased over time (first year: *M* = 3.12, *SD* = 1.23; fourth year: *M* = 3.53, *SD* = 1.56), with a standardized slope of *B* = .33 (*p* = .003), reflecting a moderate positive change relative to mathematics‐intensive subjects. This raw increase of .41 points stands in sharp contrast to the decline observed in mathematics‐intensive subjects, highlighting clear subject‐specific differences in FAB development. A slight decline was observed for biology, chemistry and computer science (first year: *M* = 2.23, *SD* = .97; fourth year: *M* = 2.19, *SD* = 1.03; *B* = .16, *p* = .013). In languages, the initial increase from the first (*M* = 2.57, *SD* = .99) to the second study year (*M* = 2.70, *SD* = 1.12) was followed by a slight decrease at the fourth study year (*M* = 2.54, *SD* = 1.09; *B* = .14, *p* = .034). No statistically significant slope difference was found for art/philosophy (first year: *M* = 2.85, *SD* = 1.18; fourth year: *M* = 2.54, *SD* = 1.16; *B* = .02, *p* = .814).

## DISCUSSION

This study had two primary objectives: (1) to examine the longitudinal development of FABs among preservice teachers over four years of university training and (2) to explore whether these developmental trajectories vary across subject groups and individual characteristics such as gender and prior academic achievement.

We hypothesized that FABs would decline over time (Hypothesis 1) and that STEM subjects would initially show stronger FABs than non‐STEM fields (Hypothesis 2a) but experience steeper decreases (Hypothesis 2b). The findings support these hypotheses, revealing a general decline in FABs over time, with mathematics‐intensive subjects initially displaying the highest FABs and social sciences the lowest. Gender differences in FABs were observed at baseline, but female and male students did not change significantly different over time. Taken together, these results highlight the subject‐specific nature of FAB development and contribute to the growing body of research on teacher beliefs about ability and intelligence.

### FAB trajectories in mathematics‐intensive subjects

A key finding of this study is that preservice teachers in mathematics‐intensive subjects, as such mathematics and physics, initially hold stronger FABs compared to those in other disciplines. This aligns with the FAB hypothesis (Leslie et al., 2015) and previous empirical findings (Heyder et al., 2019, 2020). The substantial decrease in FABs observed among these preservice teachers over time suggests that engagement in university teacher education plays a role in shifting beliefs away from an emphasis on innate ability.

Several mechanisms could explain this decline. One possibility is that preservice teachers learn to recognize the impact of sustained effort in their field, aligning with research on the positive feedback loop between achievement and beliefs about effort (Limeri et al., 2020). Additionally, exposure to the challenges of university‐level mathematics may lead them to reassess the role of talent in student success. If these students realize that excelling in school mathematics does not necessarily predict success in university‐level mathematics, they may become less inclined to attribute success solely to innate ability. These findings are particularly relevant for STEM education, where the perception of natural brilliance can shape instructional approaches and student expectations (Dweck & Leggett, 1988; Kroeper et al., 2022; Muenks et al., 2024). Despite the observed decline, it is notable that after four years of teacher education, FABs in mathematics‐intensive subjects remain higher than in most other disciplines. This persistence suggests that while teacher education may mitigate FABs, subject‐specific norms and broader cultural narratives about mathematics and innate ability remain influential. Given that mathematics is often perceived as a challenging subject with high attrition rates (Neugebauer et al., 2019), future research should explore how educational experiences (of success) shape preservice teachers' beliefs about ability and effort. The slope estimates reported in our latent growth models reflect standardized effects, as both intercept and slope factors were scaled to have a mean of zero and unit variance. While no slope coefficient was estimated for mathematics‐intensive subjects due to their role as the reference group, their raw scores showed a noticeable decline from 3.22 to 2.80 on the 7‐point FAB scale. This decrease reflects an approximate effect size of *d* = −.35, indicating a small‐to‐moderate reduction in beliefs over time—particularly notable given the typically high temporal stability of teacher beliefs (Fives & Buehl, 2012). In contrast, FABs in physical education increased by .41 scale points (from 3.12 to 3.53, *d* = .29), with the corresponding standardized slope (*B* = .33) reflecting a moderate positive change. These directional shifts underscore the importance of subject‐specific dynamics in shaping FABs during teacher education.

### Distinct trajectory of FAB development in physical education

Physical education was the only domain in which FABs increased over time, indicating a distinct trajectory compared to other subjects. While most preservice teachers developed more growth‐oriented beliefs, physical education students moved in the opposite direction, suggesting that their educational context and their learning experiences may reinforce rather than challenge fixed‐ability beliefs.

This pattern likely reflects the unique nature of physical education. The visibility of physical performance and its perceived link to success may heighten the salience of innate ability. One can also speculate that visible differences between preservice teachers in specific kinds of sports remain stable over time, which could be seen as the result of talent. Moreover, physical education students may interpret “talent” differently than peers in other academic fields, drawing on sports science discourses that emphasize talent identification and cultivation and “natural athletes” (Croston, 2012; Williams & Reilly, 2000). These messages may foster beliefs that athletic ability is innate and peaks early, reducing the perceived value of effort or training.

Importantly, what counts as “innate ability” seems to differ across disciplines. In physical education, talent often refers to visible traits like strengths or coordination, whereas in STEM it refers to abstract cognitive ability. These domain‐specific understandings of ability may help explain the divergent FAB trajectories observed and underscore the need for further research on how “talent” is conceptualized in different fields and how these beliefs shape teacher expectations and instructional practices.

### FAB trajectories in non‐STEM subjects

In contrast to STEM subjects and physical education, FABs in non‐STEM subjects—specifically languages, arts and philosophy and social sciences—started at relatively low levels and remained stable over time. This suggests that preservice teachers in these fields may already enter university teacher education with more growth‐oriented beliefs that are neither strongly challenged nor significantly reshaped during their studies.

This stability may reflect the epistemological foundations of these disciplines. Non‐STEM fields often emphasize qualities that can be cultivated through practice, such as interpretive skill or contextual reasoning (Oreck, 2004; Winner & Martino, 2000). Such growth‐oriented beliefs may be reinforced by the content and pedagogical approaches in teacher education. Prior research shows that FABs are more common in fields perceived to require unteachable brilliance (Leslie et al., 2015), and less so in domains where effort and development are seen as key to success. The stable, low FAB levels in non‐STEM subjects likely mirror these field‐specific values.

### How teacher education may influence FABs

The observed decline in FABs, particularly in STEM subjects, likely reflects the cumulative influence of several aspects of teacher education. Subject‐specific coursework may prompt preservice teachers to reflect on their own learning and recognize the role of sustained effort in mastering challenging material, especially when early confidence is challenged by university‐level difficulty. Pedagogical training (e.g., educational psychology, learning theories) often introduces growth‐oriented perspectives that encourage viewing ability as malleable rather than fixed. Practicum experiences and internships provide direct insight into student learning variability, highlighting the role of instruction, motivation and context rather than innate talent. Finally, mentoring by experienced teachers or university instructors who model growth‐oriented practices may also help shift beliefs over time.

While the current study cannot disentangle the effects of these components, the findings suggest that university teacher education has the potential to shift beliefs about ability, particularly when these beliefs are initially fixed. Future research should investigate how these elements individually and collectively shape the development of FABs across subject areas.

### Implications for teacher education

The findings of this study have significant implications for teacher education. Research has consistently shown that teachers' beliefs about ability influence their instructional practices and student outcomes. Studies rooted in mindset theory showed that fixed mindsets among educators have been linked to lower student motivation (Heyder et al., 2020) and achievement (Canning et al., 2019), while teachers with growth mindsets foster more positive learning environments (Haimovitz & Dweck, 2017) and might even help students develop a growth mindset themselves (Mesler et al., 2021).

This study provides important insights into the development of FABs at a pivotal stage in teachers' careers—their entry into and progression through university teacher education. Prior research has identified key academic transitions as critical periods for mindset development, such as the shift from primary to secondary school (Evans et al., 2018; Spernes, 2022) or during early adolescence (Mesler et al., 2021). Building on these findings, our study extends this perspective by examining how field‐specific ability beliefs evolve throughout university teacher education, highlighting this phase as crucial for belief formation and potential intervention. However, the scope of this study was limited to examining variation by subject group, gender and prior academic achievement; future work should include a broader range of contextual and instructional factors to identify mechanisms driving FAB change.

Given the subject‐specific differences in FABs, interventions to foster growth‐oriented beliefs among preservice teachers should be tailored to different disciplines. For instance, mathematics‐intensive subjects are associated with stronger FABs, suggesting a need for targeted strategies that challenge deterministic views of ability and emphasize the role of effort and learning strategies (Blackwell et al., 2007; Yeager et al., 2022). Similarly, in physical education, where the beliefs may be shaped more by perceptions of physical rather than cognitive ability, interventions could focus on instructional practices and interactions with students that reinforce effort‐oriented feedback—an approach that has been shown to support student motivation and academic achievement across disciplines (e.g., Heyder et al., 2019, 2020).

This study did not directly emphasize the question of whether talent is more crucial for success in certain fields compared to others. Prior research suggests that intelligence is a strong predictor of academic achievement across disciplines, with particularly strong effects in mathematics (Kriegbaum et al., 2018). While perceptions of ability in mathematics may have some empirical basis (Hendriyanto & Juandi, 2022; McGrew & Hessler, 1995), these beliefs can also discourage individuals—particularly those from underrepresented groups (see Rattan et al., 2012)—from pursuing careers in mathematics‐intensive fields. Thus, teacher education should balance information about intelligence as a relevant predictor for academic success with information about the potential (harmful) consequences of FABs on student outcomes.

### Limitations and directions for future research

Several limitations of the present study should be acknowledged. First, the study did not assess FABs before preservice teachers entered university teacher education. As a result, it is unclear whether the observed declines in FABs are entirely attributable to teacher education or whether they reflect broader developmental trends such as the maturity principle of personality development (e.g., Roberts et al., 2006). Against this background, future studies should include pre‐university baseline measurements to isolate the effects of university teacher education more precisely.

Second, while the study controlled for the clustered nature of the data (as teacher candidates in Germany typically study two subjects), further investigation into how these dual‐subject combinations influence FAB development would be valuable. Certain subject combinations may moderate belief trajectories, highlighting the need for more nuanced analyses. Further research should explore how specific subject combinations (e.g., STEM/STEM vs. STEM/Social Sciences) may moderate the development of FAB trajectories. German preservice teachers always study two subjects in parallel, and emerging evidence suggests these combinations potentially affect competence development and also teachers' ability belief**s** (Gildehaus et al., 2024; Mahler et al., 2021; Meyer et al., 2023). However, modelling such interactions in this study would have fragmented the sample and reduced power.

Third, while this study primarily focused on subject‐specific differences, other factors—such as preservice teachers' prior educational experiences, teaching methods (Blackwell et al., 2007; Paunesku et al., 2015), sense of belonging during their studies (Deiglmayr et al., 2019), and mentorship experiences (Yeager & Dweck, 2012)—may also shape the development of FABs. Future research should examine how these factors interact with subject‐specific beliefs to identify additional leverage points for interventions.

Fourth, a methodological limitation concerns measurement invariance of the FAB scale across academic disciplines. While the one‐factor structure and metric invariance were largely supported, scalar invariance was not consistently established, particularly at the study's beginning and end. This may affect the comparability of mean FAB scores between fields. However, partial non‐invariance is common in applied research and does not necessarily undermine group comparisons if interpreted cautiously (Kane, 2013; Robitzsch & Lüdtke, 2023).

Finally, the FAB measure itself may be interpreted differently across disciplines. While originally designed to capture beliefs about raw intellectual ability (Leslie et al., 2015), the term “talent” may evoke domain‐specific meanings, for instance, physical prowess in physical education. This semantic variation could influence how participants interpret items, potentially limiting comparability across subject groups or over time. Also, although we found little evidence for substantial variability in FAB trajectories between individuals in the present study, future research could explore whether such variability emerges under different teacher education programs, in longer time frames, or in relation to additional moderating factors such as teaching experiences or personality traits.

## CONCLUSION

This study makes several key contributions to research on teacher beliefs and the development of FABs. First, drawing on a longitudinal dataset of over 1700 preservice teachers, it represents one of the most comprehensive investigations of FAB trajectories to date. Secondly, unlike prior research focusing on single disciplines or STEM vs. non‐STEM dichotomies, this study captures the disciplinary breadth of teacher education by analysing FABs across 21 subjects, systematically grouped into six domains. Last, its four‐year design offers rare insight into how FABs evolve during a formative phase of professional development.

The combination of large sample size, disciplinary granularity and temporal depth provides robust evidence on the development of field‐specific ability beliefs during university teacher education. FABs tend to decline over time in most subject areas, especially in mathematics‐intensive fields. Yet they remain high in mathematics and physics and even increase in physical education, suggesting that subject‐specific cultures may sustain or amplify these beliefs.

These findings highlight the need to address FABs in a discipline‐sensitive manner. Interventions to foster growth‐oriented beliefs should consider the normative beliefs and professional identities tied to each subject. Helping preservice teachers develop more malleable views of ability can support their professional growth and enhance student motivation, achievement and participation in fields such as STEM.

## AUTHOR CONTRIBUTIONS

**Katharina Asbury:** Conceptualization; investigation; writing – original draft; methodology; visualization; writing – review and editing; formal analysis. **Bastian Carstensen:** Data curation; supervision; resources; formal analysis; methodology; writing – review and editing; visualization. **Uta Klusmann:** Supervision; resources.

## CONFLICT OF INTEREST STATEMENT

All authors declare no conflict of interest.

## Supporting information


Data S1.

## Data Availability

The data that support the findings of this study are available from the corresponding author upon reasonable request.
