# Supplementary material for: Changing perspectives: The development of preservice teachers' field‐specific ability beliefs across academic disciplines
Source: Br J Educ Psychol. 2025 Aug 13;96(1):337–55. doi: 10.1111/bjep.70019 (PMC12879536; doi:10.1111/bjep.70019)
Supplement: Supplementary file 1 — Data S1. [file BJEP-96-337-s001.docx]

**— Supplemental Materials —**

Changing Perspectives: The Development of Pre-service Teachers’ Field-Specific Ability Beliefs Across Academic Disciplines

Table of Contents

[Section 1: Measurement Invariance Procedures and Results 2](#_Toc202092171)

[Measurement Invariance Over Time 2](#_Toc202092172)

[Measurement Invariance Across Gender 3](#_Toc202092173)

[Measurement Invariance Across Disciplines 4](#_Toc202092174)

[Section 2: Subject-Specific FAB Development Over Time 6](#_Toc202092175)

# Section 1: Measurement Invariance Procedures and Results

## Measurement Invariance Over Time

We tested measurement invariance in FABs across the four study years using confirmatory factor analysis (CFA; Cheung & Rensvold, 2002). FABs were modelled as a latent construct with three items serving as manifest indicators. Measurement invariance was assessed by sequentially comparing increasingly restrictive models, following the step-up approach (Brown, 2006). First, we tested configural invariance, which examines whether the same factor structure holds across time, allowing all parameters (factor loadings and intercepts) to be freely estimated. Next, we tested metric invariance, which constrains factor loadings to be equal across study years, ensuring that the construct is measured consistently over time. Finally, we tested scalar invariance, which imposes additional constraints on item intercepts, allowing for meaningful comparisons of latent means over time.

Model comparisons were evaluated using multiple fit indices, including the comparative fit index (CFI), the Tucker-Lewis index (TLI), the root-mean-square error of approximation (RMSEA), and standardized root mean residual (SRMR), in addition to chi-square difference tests. Table S1 presents the results of the measurement invariance analyses.

The configural invariance model demonstrated a good fit (CFI = .99, TLI = .98, RMSEA = .02, SRMR = .04), indicating that the general factorial structure of FABs was stable across study years. Constraining factor loadings to be equal across time did not significantly reduce model fit compared to the configural model (*p*= .192), supporting metric invariance. When additionally constraining item intercepts (scalar invariance), the chi-square difference test indicated a significant decrease in fit (*p*< .001). However, other fit indices remained strong (CFI = .99, TLI = .98, RMSEA = .02, SRMR = .05), suggesting that the overall model fit was still good. Given these results, we considered scalar invariance to be largely supported and proceeded with subsequent analyses, as comparisons of latent means remained justifiable **(**for a discussion on the holistic evaluation of fit indices**, s**ee Putnick & Bornstein, 2016).

**Table S1**

*Fit Indices for Measurement Invariance Over Time for Field-Specific Ability Beliefs (FABs)*

| **Invariance Models** | | |  | **Fit indices** | | | |  | **Model comparison** | | |
| --- | --- | --- | --- | --- | --- | --- | --- | --- | --- | --- | --- |
|  | χ² | *df* |  | CFI | TLI | RMSEA | SRMR |  | ∆χ² | ∆*df* | *p* |
| Configural | 52.31 | 30 |  | .99 | .98 | .02 | .04 |  | — | — | — |
| Metric | 63.84 | 36 |  | .99 | .98 | .02 | .04 |  | x8.69 ^a^ | 6 | < .192 |
| Scalar | 118.85 | 45 |  | .99 | .98 | .02 | .05 |  | 41.85 ^b^ | 9 | < .001 |

*Note*. ^a^ Comparison of configural invariance model with metric invariance model. ^b^Comparison of metric invariance model with scalar invariance model. CFI = comparative fit index; TLI = Tucker–Lewis index; RMSEA = root mean square error of approximation; SRMR = root mean square residual.

## Measurement Invariance Across Gender

We applied the same sequence of models as used for testing invariance over time to examine measurement invariance across gender. Specifically, configural, metric, and scalar invariance models were estimated separately for male and female groups, with progressively increasing equality constraints imposed simultaneously across both groups. This involved constraining factor structures, factor loadings, and item intercepts to be equal between genders. The results are summarized in Table S2. Model comparisons were evaluated to determine whether the measurement properties of the FAB scale were equivalent for male and female participants, thereby supporting the validity of cross-gender comparisons.

Results indicated good fit for the configural model (χ²(60) = 174.99, CFI = .98, TLI = .96, RMSEA = .03, SRMR = .08), supporting equivalent factorial structure across genders. Imposing equality constraints on factor loadings (metric invariance) did not significantly reduce model fit (*p* = .429), indicating that the scale operates similarly across males and females at this level. However, constraining item intercepts to equality (scalar invariance) resulted in a significant decrease in fit (*p* < .001), suggesting some differences in item intercepts between genders. Despite this, overall fit indices remained acceptable (CFI = .97, TLI = .96, RMSEA = .03, SRMR = .08), indicating that scalar invariance was largely supported.

**Table S2**

*Fit Indices for Measurement Invariance Over Time and Gender for Field-Specific Ability Beliefs (FABs)*

| **Invariance Models** | | |  | **Fit indices** | | | |  | **Model comparison** | | |
| --- | --- | --- | --- | --- | --- | --- | --- | --- | --- | --- | --- |
|  | χ² | *df* |  | CFI | TLI | RMSEA | SRMR |  | ∆χ² | ∆*df* | *p* |
| Configural | 174.99 | 60 |  | .98 | .96 | .03 | .08 |  | — | — | — |
| Metric | 194.50 | 74 |  | .98 | .97 | .03 | .08 |  | 14.29 ^a^ | 14 | < .429 |
| Scalar | 275.82 | 95 |  | .97 | .96 | .03 | .08 |  | 62.86 ^b^ | 21 | < .001 |

*Note*. ^a^ Comparison of configural invariance model with metric invariance model. ^b^Comparison of metric invariance model with scalar invariance model. CFI = comparative fit index; TLI = Tucker–Lewis index; RMSEA = root mean square error of approximation; SRMR = root mean square residual.

## Measurement Invariance Across Disciplines

We tested measurement invariance across subject groups using the same sequence of models applied in the analyses over time and gender. However, due to convergence issues when including all measurement occasions simultaneously, invariance models were estimated separately for each time point. The results showed strong support for configural and metric invariance at all four time points, suggesting that the factorial structure and factor loadings were consistent across subject groups. At Year 2, the scalar invariance model also demonstrated a good fit, and at Year 3, scalar invariance was supported despite a slightly significant chi-square difference test (*p* = .002), with overall fit indices remaining high (CFI = .99, TLI = .99, RMSEA = .06, SRMR = .04).

However, at Years 1 and 4, scalar invariance could not be confirmed. For Year 1, model fit declined notably when intercepts were constrained (*p*< .001). Similarly, at Year 4, the scalar model showed a significant decline in fit (*p* < .001). These findings suggest that while the scale was largely invariant across disciplines at midpoints of the study, some item intercepts varied at the beginning and end, warranting cautious interpretation of latent mean comparisons across subject groups at these time points.

**Table S3**

*Fit Indices for Measurement Invariance Over Time and Gender for Field-Specific Ability Beliefs (FABs)*

| **Invariance Models** | | |  | **Fit indices** | | | |  | **Model comparison** | | |
| --- | --- | --- | --- | --- | --- | --- | --- | --- | --- | --- | --- |
|  | χ² | *df* |  | CFI | TLI | RMSEA | SRMR |  | ∆χ² | ∆*df* | *p* |
| **Year 1** |  |  |  |  |  |  |  |  |  |  |  |
| Configural | 0.00 | 0 |  | > .99 | > .99 | < .01 | < .01 |  | — | — | — |
| Metric | 5.78 | 10 |  | > .99 | > .99 | < .01 | ‍ .02 |  | 5.78 ^a^ | 10 | <‍ .429 |
| Scalar | 73.11 | 20 |  | ‍ .96 | ‍ .97 | ‍ .10 | ‍ .05 |  | 67.32 ^b^ | 10 | < .001 |
| **Year 2** |  |  |  |  |  |  |  |  |  |  |  |
| Configural | 0.00 | 0 |  | > .99 | > .99 | < .01 | < .01 |  | — | — | — |
| Metric | 14.65 | 10 |  | > .99 | .99 | .06 | .04 |  | 14.65 ^a^ | 10 | ‍ .146 |
| Scalar | 32.05 | 20 |  | .99 | .99 | .06 | .05 |  | 17.40 ^b^ | 10 | ‍ .066 |
| **Year 3** |  |  |  |  |  |  |  |  |  |  |  |
| Configural | 0.00 | 0 |  | > .99 | > .99 | < .01 | < .01 |  | — | — | — |
| Metric | 5.91 | 10 |  | > .99 | > .99 | < .01 | .02 |  | 5.91 ^a^ | 10 | ‍ .823 |
| Scalar | 33.16 | 20 |  | .99 | .99 | .06 | .04 |  | 27.26 ^b^ | 10 | ‍ .002 |
| **Year 4** |  |  |  |  |  |  |  |  |  |  |  |
| Configural | 0.00 | 0 |  | > .99 | > .99 | < .01 | < .01 |  | — | — | — |
| Metric | 28.14 | 10 |  | .97 | .99 | .10 | .06 |  | 28.14 ^a^ | 10 | ‍ .002 |
| Scalar | 67.62 | 20 |  | .96 | .97 | .12 | .07 |  | 39.49 ^b^ | 10 | < .001 |

*Note*. ^a^ Comparison of configural invariance model with metric invariance model. ^b^Comparison of metric invariance model with scalar invariance model. CFI = comparative fit index; TLI = Tucker–Lewis index; RMSEA = root mean square error of approximation; SRMR = root mean square residual.

# Section 2: Subject-Specific FAB Development Over Time

**Figure S1**

*FABs in the 21 Subjects Over the Course of University Studies*


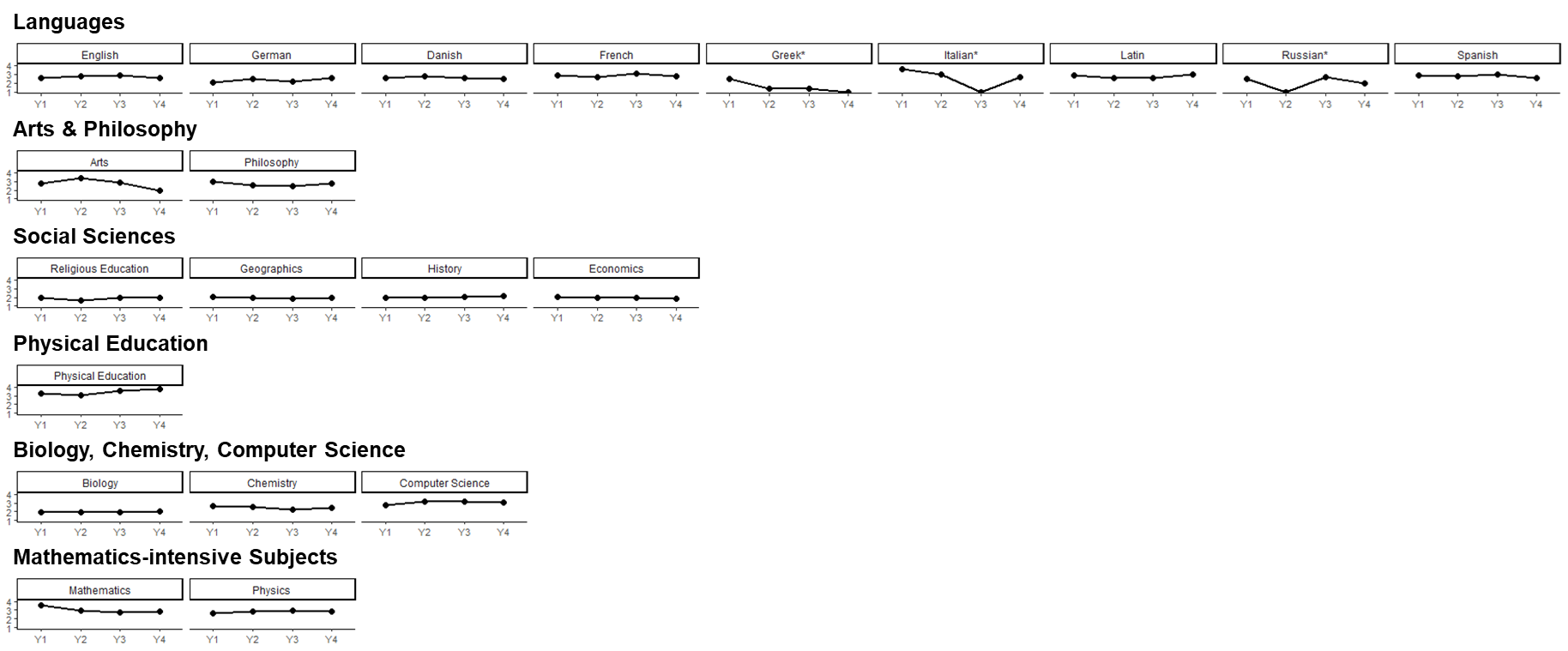


*Note.* FAB mean development in in the 21 subjects examined in this study over four study years; FAB means for subjects marked with an asterisk (*) are based on small subsamples and should be interpreted with caution; for readability, only the range from 1 to 4 is displayed.

**References**

Brown, T. A. (2006). *Confirmatory factor analysis for applied research*. Guilford Press.

Cheung, G. W., & Rensvold, R. B. (2002). Evaluating goodness-of-fit indexes for testing measurement invariance. *Structural Equation Modeling,* *9,* 233–255. <https://doi.org/10.1207/S15328007SEM0902_5>

Putnick, D. L., & Bornstein, M. H. (2016). Measurement invariance conventions and reporting: The state of the art and future directions for psychological research. *Developmental Review, 41*, 71–90. <https://doi.org/10.1016/j.dr.2016.06.004>
